# Cascara Sagrado in Constipation

**Published:** 1878-04

**Authors:** 


Cascara Sagrado in Constipation.—A Correspondent in the *Clinic* says that he has found the cascara sagrado a most admirable remedy in habitual constipation, certainly superior to any article that he has ever used. He prescribes the fluid extract. A favorite prescription of his is:

℞ Fl. ext. cascara sagrado, ℥ss.
aq. cinnamon, ℥iss.
sac. alb. ʒij.

M. S. Teaspoonful three times a day (before meals).

This is agreeable to the taste, and unlike any of the remedies heretofore employed, it soon gets the bowels into a healthy habit of action. Some patients find one teaspoonful of the mixture, that is about fifteen drops of the fluid extract at bedtime sufficient to overcome and correct the habit of the most obstinate constipation.

